# Increasing immunization coverage, Solomon Islands, 2022

**DOI:** 10.2471/BLT.24.291084

**Published:** 2024-09-02

**Authors:** Reta Angessa, Rockson Siliota, Jenniffer Anga, Tracy Kofela, Sonja Tanevska, Nemia Bainvalu, Pauline McNeil, Howard L Sobel

**Affiliations:** a World Health Organization, Office of the Representative to Solomon Islands, Ministry of Health Bldg, Chinatown Honiara, Solomon Islands.; b National and Provincial Ministry of Health and Medical Services, Honiara, Solomon Islands.

## Abstract

**Problem:**

The Malaita and Western provinces in Solomon Islands had low routine immunization coverage due to disruptions in health services caused by the coronavirus disease 2019 pandemic in early 2022.

**Approach:**

The country introduced the World Health Organization (WHO) Reaching Every District (RED) approach in 2002. Between July and September 2022, we strengthened supportive supervision, monitoring and use of data for decision-making, especially for microplanning and re-establishing outreach to prioritized areas. Health workers were supported to identify key concerns and develop strategies to improve performance. Monthly updates of reported immunization coverage, reporting completeness and fieldwork findings were widely disseminated.

**Local setting:**

Solomon Islands’ population is 748 606 people, of whom 165 345 reside in Malaita and 105 367 in Western Province.

**Relevant changes:**

In Malaita Province, reported coverage of third dose of pentavalent vaccine and first dose of measles–rubella vaccine increased from 40% (757/1892) of eligible children to 121% (1144/946) and from 30% (568/1892) to 159% (1504/946), respectively; and in Western Province reported coverage increased from 38% (443/1165) to 191% (1113/583) and from 44% (513/1165) to 149% (868/583), respectively. Reported coverage for the remaining provinces increased from 64% (3380/5282) to 88% (2325/2641) and from 59% (3116/5282) to 137% (3619/2641), respectively. These findings led the programme on immunization to re-expand the WHO RED approach nationwide.

**Lessons learnt:**

Supportive supervision, systematic monitoring and use of data for decision-making helped restoring reported immunization coverage in two low-coverage provinces. However, sustaining these results at a national level is necessary. The WHO RED approach remains relevant, even during a pandemic.

## Résumé

**Problème:**

Début 2022, les provinces de Malaita et de l'Ouest, sur les Îles Salomon, affichaient une faible couverture vaccinale due à de multiples interruptions des services de santé durant la pandémie de maladie à coronavirus 2019.

**Approche:**

Le pays a adopté l'approche «Atteindre chaque district» (ACD) de l'OMS en 2002. Entre juillet et septembre 2022, nous avons renforcé la supervision formative, la surveillance et l'utilisation de données pour la prise de décisions, en particulier dans le cadre de la microplanification et du rétablissement des programmes de proximité dans les régions prioritaires. Les professionnels de la santé ont reçu de l'aide afin d'identifier les préoccupations majeures et de développer des stratégies destinées à améliorer les performances. Les comptes rendus mensuels de la couverture vaccinale observée, le degré d'exhaustivité des rapports et les résultats des travaux menés sur le terrain ont été largement diffusés.

**Environnement local:**

Les Îles Salomon comptent 748 606 habitants; 165 345 vivent à Malaita et 105 367 dans la province de l'Ouest.

**Changements significatifs:**

Dans la province de Malaita, la couverture vaccinale observée pour la troisième dose du vaccin pentavalent et la première dose du vaccin rougeole-rubéole a augmenté, passant de 40% (757/1892) des enfants éligibles à 121% (1144/946) pour l'un et de 30% (568/1892) à 159% (1504/946) pour l'autre; de son côté, la province de l'Ouest a elle aussi connu une hausse, passant de 38% (443/1165) à 191% (1113/583) pour le premier vaccin et de 44% (513/1165) à 149% (868/583) pour le second vaccin. Dans le reste des provinces, la couverture a progressé de 64% (3380/5282) à 88% (2325/2641) et de 59% (3116/5282) à 137% (3619/2641), respectivement. Ces résultats ont abouti à un redéploiement de l'approche ACD de l'OMS pour le programme de vaccination à l'échelle nationale.

**Leçons tirées:**

La supervision formative, la surveillance systématique et l'utilisation des données pour la prise de décisions ont contribué à restaurer la couverture vaccinale dans deux provinces à faible couverture. Il est cependant impératif de maintenir ces résultats dans l'ensemble du pays. Enfin, l'approche ACD de l'OMS demeure pertinente, même lors d'une pandémie.

## Resumen

**Situación:**

Las provincias de Malaita y Occidental, en las Islas Salomón, tuvieron una cobertura de inmunización sistemática baja debido a las interrupciones de los servicios sanitarios a causa de la pandemia de la enfermedad por coronavirus de 2019 a principios de 2022.

**Enfoque:**

El país introdujo el enfoque “Llegar a todos los distritos” (RED) de la OMS en 2002. Entre julio y septiembre de 2022, se reforzó la supervisión de apoyo, el monitoreo y el uso de datos para la toma de decisiones, en especial para la microplanificación y el restablecimiento del alcance de las regiones prioritarias. Se ayudó al personal sanitario a identificar los principales problemas y a desarrollar estrategias para mejorar su rendimiento. Se difundieron ampliamente las actualizaciones mensuales de la cobertura de inmunización notificada, la exhaustividad de los informes y los resultados del trabajo de campo.

**Marco regional:**

La población de las Islas Salomón es de 748 606 habitantes, de los cuales 165 345 residen en Malaita y 105 367 en la provincia Occidental.

**Cambios importantes:**

En la provincia de Malaita, la cobertura notificada de la tercera dosis de la vacuna pentavalente y de la primera dosis de la vacuna contra el sarampión y la rubéola aumentó del 40% (757/1892) de los niños que cumplían los requisitos al 121% (1144/946) y del 30% (568/1892) al 159% (1504/946), respectivamente; y en la provincia Occidental la cobertura notificada aumentó del 38% (443/1165) al 191% (1113/583) y del 44% (513/1165) al 149% (868/583), respectivamente. La cobertura notificada para el resto de provincias aumentó del 64% (3380/5282) al 88% (2325/2641) y del 59% (3116/5282) al 137% (3619/2641), respectivamente. Estos hallazgos llevaron al programa de inmunización a ampliar el enfoque RED de la OMS a todo el país.

**Lecciones aprendidas:**

La supervisión de apoyo, el monitoreo sistemático y el uso de datos para la toma de decisiones ayudaron a restablecer la cobertura de inmunización notificada en dos provincias de baja cobertura. Sin embargo, es necesario mantener estos resultados a escala nacional. El enfoque RED de la OMS sigue siendo pertinente, incluso durante una pandemia.

## ملخص

**المشكلة:**

كانت تغطية التحصين الروتينية في مقاطعة مالايتا والمقاطعة الغربية في جزر سليمان، منخفضة بسبب الاضطرابات في الخدمات الصحية المترتبة على جائحة مرض فيروس كورونا 2019 في أوائل عام 2022.

**الأسلوب:**

قدمت الدولة أسلوب "الوصول إلى كل منطقة" (RED) التابع لمنظمة الصحة العالمية (WHO) في عام 2002. خلال الفترة ما بين يوليو/تموز وسبتمبر/أيلول 2022، قمنا بتعزيز الإشراف الداعم، والمراقبة، واستخدام البيانات لاتخاذ القرارات، وخاصة للتخطيط الجزئي وإعادة تأسيس الوصول إلى المناطق ذات الأولوية. تم دعم العاملين في المجال الصحي لتحديد المخاوف الرئيسية وتطوير استراتيجيات لتحسين الأداء. تم نشر التحديثات الشهرية الخاصة بتغطية التحصين المبلغ عنها، واكتمال التقارير، ونتائج العمل الميداني على نطاق واسع.

**المواقع المحلية:**

يبلغ عدد السكان في جزر سليمان 748606 نسمة، منهم 165345 يقيمون في مالايتا، و105367 في المقاطعة الغربية.

**التغيّرات ذات الصلة:**

في مقاطعة مالايتا، ارتفعت التغطية المبلغ عنها للجرعة الثالثة من اللقاح الخماسي، والجرعة الأولى من لقاح الحصبة والحصبة الألمانية من %40 (757/1892) من الأطفال المؤهلين إلى %121 (1144/946)، ومن %30 (568/1892) إلى %159 (1504/946) على التوالي؛ وفي المقاطعة الغربية، ارتفعت التغطية المبلغ عنها من %38 (443/1165) إلى %191 (1113/583)، ومن %44 (513/1165) إلى %149 (868/583) على التوالي. وارتفعت التغطية المبلغ عنها للمقاطعات المتبقية من %64 (3380/5282) إلى %88 (2325/2641)، ومن %59 (3116/5282) إلى %137 (3619/2641)، على التوالي. وأدت هذه النتائج إلى إعادة توسيع برنامج التحصين لنهج منظمة الصحة العالمية RED على مستوى الدولة.

**الدروس المستفادة:**

ساعد كل من الإشراف الداعم، والمراقبة المنهجية، واستخدام البيانات لاتخاذ القرارات، في استعادة تغطية التحصين المبلغ عنها في مقاطعتين منخفضتي التغطية. ومع ذلك، فإن استدامة هذه النتائج على المستوى الوطني أمر ضروري. ويظل أسلوب RED التابع لمنظمة الصحة العالمية ملائمًا، حتى أثناء الجائحة.

## 摘要

**问题:**

2022 年年初，由于 2019 冠状病毒病疫情导致卫生服务中断，所罗门群岛的马莱塔省和西部省的常规免疫接种率较低。

**方法:**

该国于 2022 年采用了世卫组织 (WHO)“达及每一地区”(RED) 的战略方法。2022 年 7 月至 9 月期间，我们加大了支持性监督、监测和基于数据进行决策的工作，尤其是在微观规划和重建优先领域的外联方面。支持卫生工作者确定关键问题并制定策略以提高绩效。广泛传播了每月最新报告的免疫接种率、报告完成度和实地调查结果。

**当地状况:**

所罗门群岛人口总数为 748,606 人，其中 165,345 人居住在马莱塔省，105,367 人居住在西部省。

**相关变化:**

据报告，在马莱塔省，符合接种五联疫苗第三剂和麻疹风疹疫苗第一剂的儿童的接种率分别从 40% (757/1892) 增加到了 121% (1144/946) 和从 30% (568/1892) 增加到了 159% (1504/946)；而在西部省，对应的免疫接种率分别从 38% (443/1165) 增加到了 191% (1113/583) 和从 44% (513/1165) 增加到了 149% (868/583)。其余省份对应的免疫接种率也分别从 64% (3380/5282) 增加到了 88% (2325/2641) 和从 59% (3116/5282) 增加到了 137% (3619/2641)。这些调查结果推动了在全国范围内进一步扩大 WHO RED 战略方法在免疫规划中应用。

**经验教训:**

进行支持性监督、系统监测和基于数据的决策有助于恢复两个低接种率省份所报告的免疫接种率。然而，有必要在国家层面维持这些成果。即使在疫情期间，WHO RED 战略方法仍具有现实意义。

## Резюме

**Проблема:**

В Западной провинции и провинции Малаита на Соломоновых Островах в начале 2022 года наблюдался низкий уровень охвата плановыми прививками из-за перебоев в работе медицинских служб, вызванных пандемией коронавирусной инфекции 2019 года.

**Подход:**

В 2002 году в стране была применена стратегия ВОЗ «Охватить каждый район» (Reaching Every District, RED). В период с июля по сентябрь 2022 года был усилен поддерживающий контроль, мониторинг и использование данных для принятия решений. В частности, это касается микропланирования и возобновления работы с приоритетными районами. Медицинские работники получили поддержку в определении основных проблем и разработке стратегий по улучшению работы. Широко распространялись ежемесячные обновленные данные об охвате населения прививками, полноте отчетности и результатах работы на выездах.

**Местные условия:**

Население Соломоновых Островов составляет 748 606 человек, из которых 165 345 проживают на острове Малаита и 105 367 – в Западной провинции.

**Осуществленные перемены:**

В провинции Малаита охват третьей дозой пятикомпонентной вакцины и первой дозой вакцины против кори и краснухи увеличился с 40% (757/1892) детей, имеющих право на прививку, до 121% (1144/946) и с 30% (568/1892) до 159% (1504/946) соответственно. В Западной провинции охват увеличился с 38% (443/1165) до 191% (1113/583) и с 44% (513/1165) до 149% (868/583) соответственно. Охват остальных провинций увеличился с 64% (3380/5282) до 88% (2325/2641) и с 59% (3116/5282) до 137% (3619/2641) соответственно. Сделанные выводы послужили основанием для программы охвата прививками вновь распространить подход ВОЗ RED на всю страну.

**Выводы:**

Поддерживающий контроль, систематический мониторинг и использование данных для принятия решений способствовали восстановлению охвата прививками в двух провинциях с низким уровнем охвата. Однако необходимо закрепить эти результаты на национальном уровне. Стратегия ВОЗ RED остается актуальной даже во время пандемии.

## Introduction

The World Health Organization (WHO) has estimated that vaccination has averted 101 million deaths in infants, globally since 1974.^1^ Unfortunately, the coronavirus disease 2019 (COVID-19) pandemic disrupted routine vaccinations, resulting in 80 million children missing at least one dose of a vaccine by May 2020, and 63 countries reporting routine immunization disruptions.^2^^,^^3^ In the Solomon Islands, two major waves of COVID-19 led to less than half of eligible children receiving the third dose of pentavalent vaccine and the first dose of measles–rubella vaccine in Malaita and Western provinces.^4^

WHO developed the Reaching Every District (RED) Approach in 2002 to provide normative guidance to reach 80% coverage for fully vaccinated children in all districts and 90% nationwide coverage. The approach consists of five operational components: (i) supportive supervision; (ii) re-establishing outreach services; (iii) monitoring and use of data for action; (iv) linking services with communities; and (v) microplanning and resource management.^5^ Here we describe how the health ministry and WHO used these five operational components to address low vaccination coverage in Malaita and Western provinces.

## Local settings

The Solomon Islands’ population is 748 606 people, of whom 165 345 reside in Malaita Province and 105 367 in Western Province. These are the largest and third largest provinces of the nine provinces, respectively.^6^

The health system is divided into hospitals, area health centres, rural health clinics and nurse aide posts. Children are vaccinated against tuberculosis, hepatitis B, diphtheria, pertussis, tetanus, *Haemophilus* *influenzae* type B, pneumococcus, rotavirus, polio, measles and rubella. Nurses and nurse aides at area health centres, rural health centres and nurse aide posts vaccinate most eligible children. The Solomon Islands has a national immunization policy and health strategic plan to guide implementation of immunization services. The health ministry introduced the RED approach in 2002, but the operational components were not implemented.^5^

The health facilities use immunization registers as the primary source of data for coverage analysis and microplanning. Health facilities report immunization data using paper-based DHIS2 forms, which are subsequently entered into an online form in the DHIS2 software at the province level.^4^

In 2021, national coverage of the first dose and third dose of pentavalent vaccines were 96% (15 912/16 575) and 87% (14 420/16 575), respectively, while the first dose of measles–rubella vaccine was 70% (11 602/16 575).^4^^,^^7^ Children aged 6 weeks or older are eligible for the first dose of the pentavalent vaccine, while the third dose is scheduled for children aged 14 weeks or older. Children aged 1 year are eligible for the first dose of the measles–rubella vaccine. For reporting purposes in the health system, children vaccinated with the pentavalent vaccine are recorded if they are younger than 1 year, whereas for the measles–rubella vaccine, the cut-off for reporting is children younger than 2 years.

## Approach

To address the decline in immunization coverage, the Ministry of Health and Medical Services convened the technical working group for immunization in June 2022. This group consisted of health ministry managers, technical experts and experts from partners, including WHO and the United Nations Children’s Fund (UNICEF). The ministry and WHO technical officers analysed DHIS2 data to identify provinces contributing the largest populations of children younger than 1 year not receiving the third dose of pentavalent vaccine between January and June 2022. They identified Malaita Province and Western Province as having the largest number of unvaccinated eligible children, 60% (1135/1892) and 62% (722/1165), respectively.^4^ The technical working group ranked the four regions of Malaita and five zones of Western Provinces based on the same criteria as above. In Malaita Province, 91% (1032) of unvaccinated children resided in two prioritized regions; and in Western Province, 86% (622) resided in three prioritized zones (Table 1). The technical working group selected 60 health facilities in the two provinces within these prioritized regions and zones for interventions to increase immunization coverage.

**Table 1:** Children not receiving third dose of pentavalent vaccine, by area in Malaita and Western Provinces, Solomon Islands, January–June 2022

Province, area	No. of children (%)
**Malaita Province (1135 children)**	
North	533 (47)
Central	499 (44)
South	80 (7)
East	23 (2)
**Western Province (722 children)**	
East New Georgia	224 (31)
West New Georgia	217 (30)
Vella La Vella	181 (25)
Shortlands	58 (8)
Central Islands	42 (6)
Rannonga/Simbo	0 (0)

Note: the pentavalent vaccine provides protection against hepatitis B, diphtheria, pertussis, tetanus and *Haemophilus* *influenzae* type B.

### Interventions

WHO and a provincial health management team provided technical and financial support for at least two supportive supervisory visits to the selected health facilities from 1 July to 30 September 2022. Using a standard checklist, the visiting team supported local health facility staff to assess routine immunization coverage, reporting completeness and quality of reports in their immunization register. Local health workers were also supported to identify problems with cold chain monitoring and stock management, by ensuring twice daily temperature monitoring of vaccine storage, and routine checks of vaccine expiration date and vaccine vial monitor status. The technical support group taught local workers injection safety, how to estimate catchment area targets and how to develop improvement plans to eliminate missed opportunities for vaccination. The improvement plan included linkage with local community groups through community health workers. Follow-up visits included a review of which parts of the plan were implemented and to identify barriers to those not accomplished. Provincial health departments provided funding, supplemented by development partners including WHO, UNICEF and the Australian Agency For International Development, where necessary.

Using DHIS2 data, the health ministry and WHO analysed the monthly immunization coverage and reporting rates per health facility, and provided written feedback to provincial health authorities, regional and zonal team leaders and health facility staff in the prioritized areas. Provincial health workers telephoned health providers when reports were delayed. 

We compared the reported vaccination coverage from January to June 2022 with that from July to September 2022, using the number of children younger than one year as the denominator for each period. This denominator was calculated by dividing the annual number of children younger than 1 year by 12, then multiplying by the number of months in the study period. Therefore, the denominator represents an estimated, rather than an exact, number of children eligible for vaccination for the study period. Furthermore, because children scheduled for vaccination in a specific month may receive vaccination later, the reported coverage for a period could exceed 100%, due to these delays. We used the same denominator to calculate measles–rubella vaccination coverage. 

## Relevant changes

**Fig. 1:**
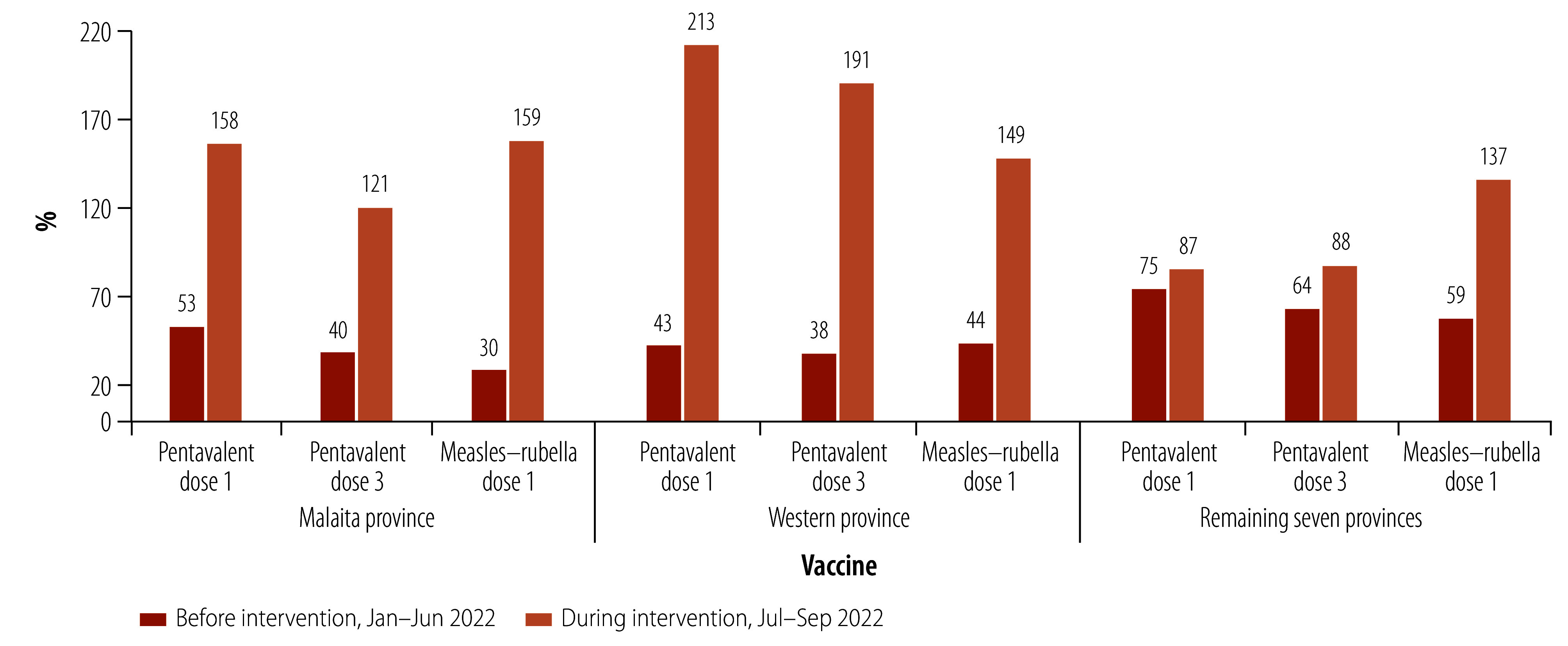
Comparative analysis of routine immunization performance before and during the intervention, Solomon Islands, 2022

Notes: the absolute numbers used to calculate the percentages are given in the text. The pentavalent vaccine provides protection against hepatitis B, diphtheria, pertussis, tetanus and *Haemophilus* *influenzae* type B.

In Malaita Province, the proportion of children reportedly receiving first and third doses of pentavalent vaccine increased from 53% (1003/1892) before the intervention to 158% (1494/946) during the intervention and from 40% (757/1892) to 121% (1144/946), respectively. A larger proportion of children also were reported as receiving the first dose of measles–rubella vaccine, from 30% (568/1892) to 159% (1504/946). Likewise, for the same period, reported coverage among eligible children residing in Western Province increased from 43% (501/1165) to 213% (1242/583) for the first pentavalent vaccine dose and from 38% (443/1165) to 191% (1113/583) for the third dose. For the first dose of measles–rubella vaccine, reported coverage increased from 44% (513/1165) to 149% (868/583). Combining reported coverage for the remaining provinces increased from 75% (3962/5282) to 87% (2298/2641) for the first pentavalent vaccine dose and from 64% (3380/5282) to 88% (2325/2641) for the third dose. For the first dose of measles–rubella vaccine, reported coverage increased from 59% (3116/5282) to 137% (3619/2641; Fig. 1).

Of 79 facilities we had data for, the number of facilities providing complete immunization reporting each month in Malaita Province increased from 38 (48%) to 56 (71%), and timeliness (report submitted on or before the specified deadline date) improved from five (6%) to 18 (23%).

Data on cold chain management and vaccine monitoring were available for Malaita Province. In the 23 facilities assessed, the number of facilities monitoring the temperature of their refrigerators twice a day increased from two (9%) to seven (30%), and facilities with a stockout of at least one vaccine decreased from 15 (65%) to 10 (43%). Additionally, facilities using either expired vaccines or vaccines beyond the discard point decreased from 15 (65%) to seven (30%). 

## Lessons learnt

The COVID-19 pandemic disrupted routine vaccinations and jeopardized global district targets of 80% coverage in two large provinces in the Solomon Islands.^5^ Here, we describe how we applied the operational components of the RED approach to increase immunization coverage in these provinces.^5^ By focusing the intervention on areas where more than three-fourths of the unimmunized children resided, we achieved an accelerated increase in reported coverage.

Because the intervention increased reported immunization coverage by one and a half to three times in the prioritized provinces, the technical working group decided to modify its national expanded programme on immunization policy in 2023. The new policy supports nationwide supportive supervision, on-the-job training and the re-establishment of outreach services, which were deemed crucial based on the lessons learnt from the field. However, further improvements in immunization coverage are needed to ensure that no child is left behind for all types of vaccinations across the country. Likewise, reporting completeness and timeliness, cold chain management, and vaccine monitoring and management all need improvement. Monitoring and supportive supervision helped clarify the degree to which these concerns were present at the health facilities.

Our findings are similar to a systematic review that showed technical support and on-the-job training for health workers were associated with an estimated 23% improvement in immunization coverage.^8^ A meta-analysis showed that multilevel interventions boosted overall immunization coverage by 25% and technical support for health workers by 13%.^9^^,^^10^

The intervention cost was 56 100 United States dollars (US$) for human resources and US$ 10 000 for fuel. However, the total economic burden per unimmunized child, including both direct medical costs and productivity losses, is estimated to be US$ 800–US$ 1400 in low- and middle-income countries.^11^^–^^13^

Implementing the RED approach had several challenges. Incomplete immunization data hampered accurate estimations of coverage and the identification of unimmunized children. Common issues with immunization data included double counting, underreporting and late reporting. Furthermore, the use of a set estimated denominator led to reported coverage above 100%. In the Solomon Islands, unreliable mobile networks further complicate reporting. The absence of a sufficient budget for fuel and daily allowances restricted regular supportive supervision, the re-establishment of outreach services by local health facilities and the delivery of vaccines to health facilities. In addition, a few health workers had difficulty with numeracy, rendering monitoring and use of data for local action difficult. The WHO and health ministry team supported solving these challenges through on-the-job training for the prioritized health facility staff on data management, reminder calls to health facilities with late reporting, feedback to provincial authorities, and coordinating with partners supporting the immunization programme to reduce budgetary constraints. The main lessons learnt are presented in Box 1.

Box 1 **Summary of main lessons learnt**

- Prioritization of areas based on number of unimmunized children rapidly improved immunization coverage
- Low immunization coverage caused by disruptions from outbreaks can be improved by implementing the Reaching Every District (RED) approach
- Supportive supervision and monitoring and use of data for action were crucial for targeted microplanning and re-establishing outreach services to restore immunization efforts.

With all vaccination programmes, sustained and expanded support is needed to maintain high coverage in Malaita and Western provinces, while improving reported coverage in other provinces. Many countries will likely benefit from implementing the RED approach, specifically systematic monitoring and use of data for action, analysis and feedback of facility-level reported data, re-establishing outreach services and supportive supervision with on-the-job training.
